# Supporting a ‘good life’ for autistic children: Autistic adults’ and parents’ perspectives

**DOI:** 10.1177/13623613261418945

**Published:** 2026-02-18

**Authors:** Kate Simpson, Connie Allen, Elizabeth Wheeley, Elizabeth Pellicano, Melanie Heyworth, Jacquiline den Houting, Rachael Bowen, Dawn Adams

**Affiliations:** 1 Griffith University, Australia; 2 Macquarie University, Australia; 3 University College London, UK; 4 Reframing Autism, Australia; 5 La Trobe University, Australia; 6 Giant Steps, Australia

**Keywords:** autism, children, qualitative research, quality of life, wellbeing

## Abstract

There has been limited research into what helps to promote autistic children’s quality of life. This qualitative study, co-produced with autistic people, aimed to identify, from multiple perspectives, what helps autistic children to live a ‘good life’. We conducted semi-structured interviews with autistic adults (*n* = 28) and parents of autistic children (*n* = 29). Using reflexive thematic analysis, we identified four themes: *Being accepted by others in a way that allows the child to be themselves; Finding ‘the things that light [the child]’; Having a sense of control over their own life* and *Physical/sensory environments matter.* All themes were common to both autistic adults and parents of autistic children, with the exception of one sub-theme, which was predominantly driven by autistic adults’ responses. These findings highlight potential pathways to support autistic children’s quality of life now and into the future.

**Lay Abstract:**

There hasn’t been much research about what helps autistic children to live a ‘good life’ or to have a good quality of life. This makes it difficult to know how to help autistic children to do so. In this study, we asked autistic adults and parents of autistic children what they thought helped autistic children to live a ‘good life’. They mentioned four important areas. These were (a) autistic children being accepted as their real or true self, (b) autistic children finding the things that they enjoy and that energise them, (c) autistic children having a sense of control in their life and (d) physical environments that suit autistic children’s sensory needs. These are things that can be changed in the autistic child’s environment to support the autistic child in living a good life.

Quality of life (QoL) is a multidimensional construct that describes a person’s wellbeing or satisfaction with their life across multiple domains, including physical, mental, emotional and social functioning (Chezan et al., 2022; Evers et al., 2022; The WHOQOL Group, 1995). Each individual’s QoL is shaped by their personal characteristics, their environment/s and the interaction between these personal and environmental factors. QoL can be considered to comprise both objective (i.e., observable indicators; what a person’s situation *is*) and subjective (i.e., individual perception; what a person *thinks* or *feels* about their situation) components (Coghill et al., 2009; Evers et al., 2022). Importantly, people may vary in the subjective value they ascribe to various QoL domains; as a result, what constitutes ‘good’ QoL may differ from person to person.

Research examining autistic QoL has typically relied on normative conceptualisations, assessing QoL using quantitative measures developed for the general population (Evers et al., 2022; Simpson et al., 2024). Such studies typically report lower QoL scores for autistic participants compared to non-autistic controls (e.g., Egilson et al., 2017). However, the more recent literature identifies that the use of ‘other-defined’ measures of QoL (i.e., those designed by and for non-autistic people) fails to capture aspects of QoL that might be meaningful or important to autistic people (Pellicano et al., 2022; Pellicano & Heyworth, 2023). Such approaches overlook neurodevelopmental differences and provide limited insight into autistic experiences of life. Similarly, common QoL measures often primarily assess physical and emotional wellbeing, with a relative underrepresentation of items assessing other key QoL domains including self-determination, social inclusion and rights (Evers et al., 2022). While some researchers have attempted to address this gap by incorporating autistic perspectives into existing QoL models (e.g., McConachie’s add-on for the World Health Organization Quality of Life Brief Version [WHOQoL-BREF]; The WHOQOL Group, 1998), others (e.g., Lam et al., 2021; Pellicano et al., 2022; Pellicano & Heyworth, 2023) go further to suggest that using normative accounts of QoL necessarily constrains our understanding of what a good life means to autistic people. In their review of QoL in autistic adults, Pellicano et al. (2022) settled on Martha Nussbaum’s (2000) Capabilities Approach which allows us to identify key dimensions of a flourishing life for any human being – such as physical health or connections with the natural world – but leaves the *content* of each of those dimensions flexible enough to respond to the particular needs and desires of people themselves. Identifying strengths and challenges within a Capabilities Approach shifts the focus from measuring outcomes to enabling opportunities for a good life as defined by autistic people themselves. This complements the neurodiversity-affirming perspective which acknowledges that cognitive variation is part of human diversity, and that disability arises from the interaction between an individual and the surrounding environment (Dwyer, 2022).

In-depth, qualitative methods enable interrogation of potentially non-autistic assumptions and biases and elucidation of autistic and autism community perspectives regarding what is (and is not) important in achieving good QoL (Paricos et al., 2024; Pellicano et al., 2022). In a recent interview study examining what good QoL means for autistic people (Merrington et al., 2024), autistic participants (34 adults and 14 adolescents) described two themes that they felt supported a good life: ‘feeling good’, encompassing enjoying yourself, social connections and safe sensory environments; and ‘being myself in the world’, including self-acceptance, agency, meaningful work and access to support services. Being able to live a good life was hindered by feelings of ‘exhaustion and overwhelm’ associated with navigating daily life, and negative encounters with others, which led to poor self-perception (Merrington et al., 2024). This study, aligned with a capabilities approach (Pellicano et al., 2022), provides valuable insight to support QoL in autistic adults and adolescents. There is limited research, however, that has examined how a good life for autistic children can be facilitated, and it cannot be assumed that what supports wellbeing in adolescence or adulthood will be the same in childhood; indicators of QoL must be applicable to children and sensitive to developmental changes (Wallander & Koot, 2016). In three recent studies, autistic adults provided reflections on childhood experiences via survey (Lee et al., 2023) and interview (Øverland et al., 2024; Lichtlé et al., 2022) methods. Broadly, they highlighted the need to embrace the child’s autistic identity; show acceptance and unconditional love; understand and meet the child’s uniquely autistic needs; provide structure, predictability and safety; value the child’s interests; support social and emotional connections; and prioritise parent wellbeing and advocacy.

In this study, we sought to build on this work by eliciting perspectives of autistic adults *and* parents of autistic children, to expand understanding of QoL in autistic children. Qualitative investigations of autistic QoL (in children, adolescents and adults) have tended to rely on data from single informant groups – either autistic people or parents of autistic children (Epstein et al., 2019; Lee et al., 2023; Lichtlé et al., 2022; McConachie et al., 2020; Merrington et al., 2024; Øverland et al., 2024) – and the same goes for quantitative investigations (Evers et al., 2022), despite evidence of discrepancies between informants (see De Los Reyes & Epkins, 2023). The use of multiple groups of informants to explore dimensions impacting autistic children’s QoL provides the opportunity to explore converging and diverging factors which may be specific to situations or positionality. Specifically, we asked, *What may help (or hinder) autistic children’s QoL, from the perspectives of autistic adults and parents of autistic children?*

## Method

Ethical approval was granted by Griffith University Human Research Ethics Committee (HREC 2022﻿/783) prior to commencing this study.

### Participants

Australian autistic adults and parents of autistic children (referred to as *adults* and *parents*, respectively) were recruited through research centre and community organisations’ social media posts. In total, 57 participants, including 28 adults (49%) and 29 parents (51%), completed interviews (see Table 1). Most participants described themselves as white and female. Twenty-two (39%) participants were autistic parents of autistic children; seven chose to report on factors influencing a good life for their autistic child (thus participating as parents) and 15 reported on their own childhood experiences (therefore participating as adults).

**Table 1.** Participant and child characteristics.

	Total (*N* = 57)	Autistic adults (*n* = 28)	Parents of autistic children (*n* = 29)	Autistic children reported on (*n* = 29)
Age mean in years (range)	42 (18–65)	39 (18–65)	44 (29–64)	10 (3–20)
Sex/gender identity				
Female	49 (86%)	21 (75%)	28 (97%)	10 (34%)
Male	5 (9%)	4 (14%)	1 (3%)	19 (66%)
Non-binary or gender questioning	3 (5%)	3 (11%)	0	
Ethnicity				
White Australian	54 (95%)	28 (100%)	26 (90%)	
Asian Australian	1 (2%)	0	1 (3%)	
Not provided	2 (3%)	0	2 (7%)	
Autism diagnosis				
Formally diagnosed	27 (47%)	23 (82%)	4 (14%)	29 (100%)
Self-identified (no formal diagnosis)	8 (14%)	5 (18%)	3 (10%)	
Not diagnosed or self-identified	22 (39%)	0	22 (76%)	
Co-occurring conditions (formally diagnosed)				
Attention deficit hyperactivity disorder		10 (36%)		9 (31%)
Anxiety		10 (36%)		7 (24%)
Depression		7 (25%)		2 (6%)
Medical conditions (e.g., epilepsy, fibromyalgia)		6 (21%)		1 (3%)
Post-traumatic stress disorder		5 (18%)		-
Speech language disorder		-		5 (17%)
Intellectual or developmental disorder		-		1 (3%)
Other (e.g., dyslexia, chronic fatigue, obsessive-compulsive disorder)		5 (18%)		2 (6%)

The numbers refer to the mean (range) or *n* (%).

Parents reported on the formative experiences of 29 autistic children (*M*_age_ = 10 years, range 3–20 years at the time of the interview), all of whom had received a formal autism diagnosis. Where parents had more than one autistic child, they were asked to report on one child only. For those with older children, they were invited to reflect on their child’s experiences growing up. Seventeen (61%) of the participating autistic adults and 11 (38%) autistic children were reported to have at least one co-occurring condition (see Table 1).

### Interview procedure

Semi-structured interviews were conducted by one of two interviewers: an autistic researcher experienced in interviewing and a non-autistic researcher with experience interviewing autistic people. Four parents and seven adults specifically requested (and received) an autistic interviewer. All interviews were conducted using online video conferencing software, with questions emailed to participants in advance. At the start of each interview, participants were advised that they were welcome to move, stop, take a break and/or respond in writing through the chat function as required and to leave their cameras off if preferred.

The interview questions (Supplementary File) were developed collaboratively by the researchers and representatives from community groups to explore factors that help autistic children to ‘live a good life’. The phrase *live a good life* was recommended by our autistic and parent advisory group, as they felt it was more accessible and understandable to the community than ‘QoL’. Participants were asked to describe what a good life entailed for them as children (adult participants) or for their autistic child (parent participants). They were asked to identify facilitators and barriers to achieving a good life across five domains: staying healthy, having fun, learning, family and friends, and having a say/feeling in control. These domains were informed by Pellicano et al. (2022), who drew upon Nussbaum’s (2000, 2011) capabilities approach. Finally, participants were asked to identify one thing they would change to enable a good life for all autistic children.

Interviews ranged from 31 to 165 min (adults *M* = 81 min; parents *M* = 69 min) and were audio recorded and professionally transcribed. Participants were able to check their deidentified transcripts and request changes, which resulted in minor alterations (e.g., clarifying information and redacting some information). Participants were provided with a $50 AUD voucher to thank them for their time and expertise.

### Data analysis

We employed reflexive thematic analysis with an inductive approach, using Braun and Clarke’s (2022) iterative, six-phase process. Data were analysed at the semantic level, with transcripts coded based on the explicit meaning of participants’ words, powered by NVivo (QSR International, 2020). In early stages of analyses, parent interviews and autistic adult interviews were treated as two discrete data sets. Each data set was analysed by a researcher focusing solely on their participant group. Each coder kept a reflexive journal, where they recorded their personal responses to and interpretations of the data. On a regular basis, they individually met with the lead author and discussed their reflections and coding. This enhanced confirmability to ensure that the researchers’ interpretations were derived from intimate knowledge of their data set while strengthening dependability of findings through ensuring that the processes were logical and clearly documented. These researchers were experienced in qualitative research, with deep understanding and/or lived experience.

Preliminary discussions with the project team (see section ‘Community involvement’) revealed common themes across adult and parent data. As a result, over several meetings, themes from both groups were compared, merged, defined, refined and the final themes named. During this process, we considered how team members’ positionality (e.g., professional backgrounds, academic vs. clinical backgrounds and professional vs. lived experience of autism) might influence our views about the interpretations of the data and inform resultant codes and themes. For example, different ideas about the degree to which acceptance (Theme 1) related to other themes and sub-themes were discussed and resolved within the team. The final stage involved drafting the manuscript, with all authors reviewing and refining the results. Trustworthiness was enhanced through prolonged engagement with the data, reflexivity and sharing of analytical decisions with the project team throughout the study (Nowell et al., 2017).

### Data integrity issues

Like in other studies (e.g., Pellicano et al., 2023), the initial recruitment process resulted in several ‘scammer participants’. These participants were identified during interviews on the basis that they (a) did not reside in the country of focus (Australia) and (b) demonstrated limited understanding of autism. Recruitment was halted, and systematic strategies (see Pellicano et al., 2023) were implemented to assess participant integrity prior to interview. After implementation of these procedures, recruitment was resumed, with no further concerns regarding participant or data integrity. Interviews with suspected scammer participants were not transcribed or included in this study.

### Community involvement

The project team includes academic (autistic, non-autistic neurodivergent and neurotypical researchers) and community (representatives of autistic-led and other community organisations) partners. The academic and community partners were involved in the grant application, study design, collection and analysis of data, and manuscript preparation and review and are named as co-authors on the paper. Monthly meetings included all academic and community team members. To support accessibility and accountability, there were multiple methods for engaging with project materials and for providing input, with sufficient time provided for all team members to contribute. The project was led by a non-autistic, neurodivergent, established academic who, by nature of their role, held final decision-making power. Nonetheless, we attempted to minimise power imbalances; for example, where possible, the team made decisions collaboratively during monthly project meetings.

## Results

Based on participants’ responses, four themes are reported. Theme 1 – Being accepted by others in a way that allows the child to be themselves – included two sub-themes: 1.1. Making positive connections and 1.2. The detrimental impact of not being accepted or included. The remaining themes were Theme 2 – Finding ‘the things that light [the child] up’, Theme 3 – Having a sense of control over their own life and Theme 4 – Physical and sensory environments matter. Each theme represented the views of both autistic adults and parents, except for Sub-Theme 1.2, which predominantly came from adults’ data. Quotes are attributed using alphabetic codes, with the preface ‘A’ indicating adults and ‘P’ indicating parents.

### Theme 1. Being accepted by others in a way that allows the child to be themselves

Adults and parents both described acceptance as the foundation for autistic children to live a good life. Acceptance included being ‘loved’, ‘understood’, ‘appreciated’ and ‘respected’, without having to ‘sacrifice who you are as a person’ (A_SC). Participants explained that children need to feel comfortable to be themselves, without others ‘pushing against it’ (P_XU) or facing ‘an expectation that you [have] to put on a persona’ (A_GQ). According to one parent, this meant not trying to ‘put him in a box . . . If he does something differently, so what?’ (P_AX). Adults explained how people need to understand children’s ‘little quirks’ (A_ZJ) and embrace autistic children’s individuality, even if it ‘sits a long way from typical’ (A_TD). One parent advised autistic children to ‘be their authentic selves’, as ‘their way of being is the right way for them’ (P_RO). This acceptance helped children to realise ‘you’re okay to be who you are’ (A_TD), leading them to ‘blossom’ (A_JT).

#### Sub-Theme 1.1. Making positive connections

Being accepted enabled autistic children to develop positive connections with others, including peers, family members, people in the community and teachers. Fostering positive connections involved people ‘listening’ to and believing the child (A_KU) and letting them be themselves without ‘judgement’ (A_GQ). These connections provided children with a sense of ‘freedom’ to be themselves (A_FP) and also created a sense of belonging and enjoyment. For one parent, this meant her child’s best friends were children who ‘really get him and love him’ (P_PM). For another, it meant their child felt ‘part of the gang’ and ‘he loves going there . . . he’s accepted’ (P_DA).

Adults and parents described how positive connections may look different for autistic children compared to non-autistic children. One parent explained that it was important to ‘figure out what social is for [the child], because it doesn’t have to be the way it is for other people’ (P_RO). Participants described positive connections that involved playing ‘with people in his own time’ (P_CZ), ‘both doing a thing in the same room and not necessarily interacting a lot’ (i.e., parallel play; A_NX) and sending funny Instagram reels in an asynchronous manner (A_EO; P_UR). One parent described how her child connected with children online through her interests, reflecting that she ‘seems happy with the way her social life goes’ (P_XU).

Parents also spoke about recognising their child’s connections with them. For example, one parent described her child as ‘a people person’ who ‘just wants to play . . . wants to interact . . . wants to be around you’ (P_EB). Finding ways to connect was also a consideration, with a parent explaining:

He’s a helper, so he helps me with the shopping . . . or if we’ve got any jobs to do, he’ll come . . . it’s just a nice way for us to do something together and connect because sometimes it’s hard to find those ways to connect. (P_MJ)

Connections with other autistic people were also considered important, but these may take time. For example, one parent described her daughter’s experience, saying, ‘she’s slowly finding her tribe with other autistic friends’ (P_XU). For others, they may still be trying to find connections with autistic people. One adult expressed sadness that her parents were still her ‘only friends’, explaining that ‘I do things on my own because I have yet to find a tribe who want to participate in [my] hobbies’ (A_WG).

#### Sub-Theme 1.2. The detrimental impact of not being accepted or included

Adults shared in detail the impact of not being accepted. They spoke about feeling shunned and excluded, describing this ‘pain’ as impacting ‘every aspect of your life’ (A_CM). Another adult explained, ‘if you feel like an outcast and you don’t know what the rules of the game are . . . you’re never going to be happy’ (A_MW). Three adults used the word ‘gaslit’, describing how their interactions with others led them to doubt themselves and to trust others’ judgements and opinions over their own. For one adult, knowing that he was different from other children without knowing why, or how to fit in, had a deep impact: ‘you’re looked at [with] contempt . . . you’re not trying to be different. You just are. You feel worthless’ (A_XH). Being viewed as different often led to autistic children being bullied, which negatively impacted wellbeing. As A_YI noted, ‘you can’t live your best life when you are constantly feeling vulnerable’.

Many adults described trying to fit in during childhood by hiding their differences. This, however, was also detrimental. As one adult warned, trying to fit in may mean ‘hacking away at [your] identity’ (A_SC). Another adult described her younger self as ‘literally just a mirror’ to the people around her:

I was their personality, their sense of humour, their energy level . . . I just would copy it and I’d send it straight back at them. So I basically had no identity . . . I was constantly exhausted . . . I just got super burnt out. (A_EO)

Consistently conforming to neurotypical expectations, according to one adult, ‘gets in the way of figuring out who you are’ (A_FP).

### Theme 2. Finding ‘the things that light [the child] up’

Both adults and parents identified finding and engaging in enjoyable activities as key for autistic children to live a good life. One adult recommended that children find ‘the things that light you up [and] make you feel energised’ (A_FP), something they had not been able to do as a child. Other adults reflected on their own positive childhood experiences of ‘wading in the waters of [your] passion’ (A_VF) or engaging in the ‘quiet time’ that brought them ‘a lot of joy’ (A_RB). These activities provided opportunities to understand and express themselves, to make positive connections (Sub-Theme 1.1) and to achieve success. As one parent said, ‘we kept her extracurricular [activities] up because they were the only thing giving her a measure of success’ (P_KH). In addition, enjoyable activities supported children’s mental wellbeing, helping the child to ‘recharge’ and ‘stay mentally healthy’ (A_DN), to ‘decompress’ (A_NX) and ‘escape’ (A_KA) or to look forward to something when coping with challenges (A_OY). P_OL said, ‘If he could do what he likes doing, he would be living his best life’.

Finding a child’s interests may take time and exploration. One parent explained that their child benefitted from ‘allowing him to explore what’s best for him and . . . learn [in] whatever way he learns and encourage that’ (P_AX). Naturally, interests vary for each person. Adults recalled enjoying childhood activities including building cubby houses with friends (A_LB), spending time alone on a farm (P_KH), Girl Guides (A_IS) and playing basketball (A_LB). Similarly, parents described the diverse range of activities their children enjoyed. One spoke of her son’s love for the sea: ‘He will walk for hours on the beach. He loves the waterline, loves the sensation of the sand’ (P_AX). Another described how spending time in the garden, ‘watching the magpies, catching skinks’ (P_UR) brought her child joy, while a third child enjoyed creating things and pretending to be a superhero, but ‘his favourite thing was making his sister laugh’ (P_QN). Other children enjoyed organised activities: ‘loves’ drama and art, participating ‘enthusiastically’ (P_OL), while another selected martial arts because ‘she’s into Lego Ninjago . . . she wanted to be a ninja’ (P_XU). Ultimately, to promote QoL, it was important to identify activities that the child valued and to foster these interests, as one parent summarised:

When she’s on her computer games, she has a headset on, she’s got music playing in the background, and you just look at her and you just know she’s in the happiest place in the world. That’s really important to her. (P_UR)

While ‘finding things that light [the child] up’ was a distinct theme, acceptance (Theme 1) played a vital role in enabling children to shine. Participants pointed out that autistic children may have interests ‘that other people don’t [or can’t] understand’ (A_EO) and highlighted the need to accept these interests, because ‘fun . . . can look different’ (A_FP) and ‘just because it’s different, doesn’t mean it’s wrong’ (P_RO). One parent felt that ‘if we just let them follow their brilliance . . . this is where some of the most amazing people on this planet have come from’ (P_DA).

### Theme 3. Having a sense of control over their own life

Adults and parents agreed that being able to make choices about their own lives, and having these choices respected by others, could enable autistic children to live a good life. Reflecting on childhood, adults spoke about the freedom they experienced when they were able to make their own choices. For one adult, ‘feeling in control [was] absolutely essential’ for autistic children to live a good life (A_DN). Both adults and parents identified that children should have autonomy over decisions including what they wear, eat and learn; being able to say ‘no’; avoiding unwanted touch; and deciding when to disclose their autistic identity.

While adults advocated for autistic children to ‘take control of aspects of their lives’ (A_EO), parents highlighted the complexities of such autonomy, noting that this may require children understanding their options, being given the opportunity to exercise choice-making and having their choices respected. One parent noted that decision-making abilities may vary with age, explaining that children should have ‘age-appropriate choice’ (P_TQ). Some parents facilitated opportunities for their child to exercise their right to choose. For example, one family had ‘crafted’ a way for their child to indicate waning capacity in social settings, by whispering ‘Mummy, I feel peopled out’ (P_VS). Another parent selected activities that allowed her child to choose her level of involvement:

It might be we’ll go to the beach; she can go off on her own and she can look for shells or she can come back, and she can hang with the other young person, and they can build a sandcastle together. But it’s again that choice. (P_VS)

### Theme 4. Physical and sensory environments matter

Parents, in particular, discussed how comfortable physical environments can support a good life. Parents explained that when their child felt comfortable in an environment, they were more likely to enjoy activities. For example, P_FC talked about how her child could enjoy going to the park when it wasn’t too busy, saying, ‘you can just see how much fun he’s having and enjoy it’; conversely, he would withdraw when the park was busy. Comfortable environments also included places the child could use when they needed their ‘own space’ to ‘have a rest’ and ‘time to relax’ (P_AD). To support this, one family set up their home to meet their child’s need for soft sensory experiences: ‘we have our entire house full of soft blankets and pillows. We have a crash mat, we have a sensory swing. Those things are what helps make her day better’ (P_GD). Finding environments outside the home that met children’s needs required planning by the parents. P_TQ spoke about carefully planning activities to avoid ‘an overstimulating environment’ with ‘lots of change’ to ‘set [their child] up for success’. Despite planning, though, finding comfortable environments could be challenging. P_HE, for example, relayed the difficulty of finding a quiet space ‘where there’s not a speaker blaring at you’ so that her child could enjoy a theme park.

Both adults and parents identified that a lack of access to these safe and secure physical spaces could induce anxiety and overwhelm, negatively impacting children’s QoL. Adults recalled how unmet sensory needs in childhood led them to believe that ‘the world [was] a scary place’ (A_PZ) and described how sensory stressors impacted their physical health; one adult, for example, recalled becoming ‘vomity’ in uncomfortable temperatures (A_KA), while others reported migraines induced by lights (A_PZ), sounds (A_DN), smells (A_QA) and social gatherings (A_HR). Parents recognised that their children faced similar challenges: ‘He has to spend a lot of time working very hard to be in spaces that he’s not comfortable in, and that’s such a massive strain’ (P_OL). Comfortable physical environments, in contrast, enabled children to feel ‘calm, safe and regulated’ (P_EB).

## Discussion

In this study, we interviewed 57 autistic people and parents of autistic children to identify the dimensions they perceived as leading to good QoL for autistic children, addressing a noted gap in the literature (Najeeb & Quadt, 2024). Gathering data from multiple groups of informants extends knowledge by integrating distinct perspectives, which may be specific to situations or positionality (De Los Reyes & Epkins, 2023). In this study, parents provided an outsider view of their child’s current context, while autistic adults provided retrospective firsthand insights. Including both groups offers complementary viewpoints, and the convergence of their perspectives strengthens the trustworthiness of the findings. The four themes identified by both groups reflect capabilities required for autistic children living a good life. These include being able to be their authentic self and form connections in ways that suit them; engage in preferred activities on their own terms; make decisions and exercise control over their lives; and access and feel safe and comfortable in their environment. The findings identify foundational capabilities within a neurodiversity-affirming framework.

Our findings align with recent literature reporting on factors supporting autistic QoL. Previous research has identified understanding of autism as impacting autistic people’s QoL (McConachie et al., 2020; Øverland et al., 2024). Our findings extend this to highlight unconditional acceptance as foundational to living a good life. For children, their family is their immediate environment, making family acceptance especially important. Lee et al. (2023) identified the importance of parents demonstrating unconditional love and acceptance as a key component of positive parenting experiences reflected by autistic adults. This acceptance allows parents to focus on ‘their child’s happiness, rather than neurotypical success’ (Lee et al., 2023) and enables the autistic person to be themselves in the world (Merrington et al., 2024). The connection between acceptance and QoL is multifaceted. Being accepted by others supports the development of self-acceptance (Davies et al., 2024), which itself is underpinned by the development of positive self-identity as an autistic person (Cooper et al., 2023) – a factor that has been identified by autistic adults as missing in neurotypical QoL measures (McConachie et al., 2020). The detrimental impact of not being accepted (Sub-Theme 1.2) can lead to internalised stigma (Botha & Frost, 2020; Huang et al., 2023), feelings of helplessness associated with being autistic (Nguyen et al., 2020) and negative QoL (Epstein et al., 2019).

In our own study, acceptance was foundational for making positive connections with people who ‘get’ them (Sub-Theme 1.1), suggesting that it is important to consider acceptance both within and beyond the family, including acceptance by peers. Social connections are identified in neurotypical QoL measures and have been identified as a factor supporting autistic QoL (Epstein et al., 2019; Lichtlé et al., 2022; Øverland et al., 2024). However, our data emphasise that positive social connections are dependent on accepting the autistic child’s degree and method of connecting. This is a nuance that is not captured in conventional QoL measures (Epstein et al., 2019), yet it is recognised as a capability for supporting autistic adults to live a good life (Pellicano et al., 2022). To support positive connections for autistic children means parents recognising and accepting the nature of their child’s friendships, which may differ from neurotypical development in terms of number of friends and ways of interacting (Fox et al., 2024). This recognition is equally as important for professionals, where the focus of friendship interventions continues to be on developing the autistic child’s social skills to align with neurotypical expectations (Chang & Dean, 2022; Micai et al., 2024).

Adults and parents described meaningful participation in enjoyable activities as supporting autistic children’s QoL (Theme 2), adding to a growing body of research documenting the importance of autistic children engaging in their interests (Lee et al., 2023; Lichtlé et al., 2022). This factor was also identified as important for adults in the capabilities approach (Pellicano et al., 2022). Children’s interests could be solitary or could facilitate social connections and spanned a diverse range consistent with interests previously described by autistic adults (Grove et al., 2018). Participation was enabled when children had opportunities to try different activities and were supported to engage in interests and activities of their choosing – even when these choices were unconventional.

Indeed, both adults and parents highlighted the need for autistic young children to have the opportunity to make decisions and have a sense of control over their lives (Theme 3). Participants identified the importance of empowering autistic children to make choices about how, where and with whom they spend their time; to solve problems; to be listened to; and to have their perspectives acknowledged and accepted. Supporting autistic children to have agency in their life may require not only providing opportunities for them to share their preferences or needs but also offering different modes for them to express themselves and building the skills needed to do so. The association between autonomy and QoL in autistic people has been documented in studies with autistic adolescents and adults (Najeeb & Quadt, 2024), but this is one of the first studies to report this association for autistic children. As Pellicano and Heyworth (2023) stated, ‘If we want to take wellbeing seriously, we need to take Autistic autonomy seriously too’ (p. 421); our findings suggest that this holds true even in the early years.

Last, our participants described the value of safe and comfortable environments in supporting autistic children’s QoL. Although the relationship between the physical environment and QoL in autistic children (Theme 4) is reported in the qualitative literature (Lee et al., 2023; Lichtlé et al., 2022), it was not identified in a systematic review of quantitative research exploring the predictors of health-related QoL in neurodivergent children (Mahjoob et al., 2024). This indicates a gap in the understanding of environmental influences, such as the impact on QoL of the built environment, access to care and resources, accommodations, as well as other factors that impact the person–environment fit (Pellicano et al., 2022). Given that these have been highlighted in numerous qualitative studies and are often the focus of supports or accommodations put into place to support young children (Erez & Gal, 2020), more research is needed to further understand their role in QoL in autistic children. While our participants primarily spoke of environmental factors in terms of sensory impact, both physical (sensory) and social (attitudes) environments have previously been identified as facilitating – or impeding – autistic children’s participation in activities in the home and the community (Simpson & Adams, 2023).

Supporting autistic children to live a good life, both now and into adulthood, requires the adoption of a neurodiversity-affirming approach that respects individual differences in what a good life may look like. Previous research (see Sáez-Suanes & Álvarez-Couto, 2022) has identified several modifiable factors associated with improved QoL for autistic adults, including social participation, autonomy and empowerment, and engagement in leisure activities, while sensory hypersensitivity has been negatively associated with QoL.

This work aligns with the findings reported in our study for autistic children and those of prior qualitative studies that have explored dimensions of autistic QoL across different age groups (Lichtlé et al., 2022; Merrington et al., 2024; Øverland et al., 2024). These studies consistently highlight the importance of acceptance, personal interests, social connections and safe environments. Our findings add to this by highlighting that dimensions such as acceptance, personal interests, social connections and safe environments may be foundational from early childhood and continue to shape wellbeing across developmental stages. While these features remain important across the lifespan, their expression and significance may shift with age and development. Taken together, these findings underscore the need for neurodiversity-affirming services that support families and inform professional practices. Such services should be designed to foster environments that enable autistic children to thrive, with a focus on promoting wellbeing, inclusion and self-determination from an early age.

However, these dimensions are not discrete, as evident in this study. The complexity of and interaction between dimensions has rarely been explored in the quantitative literature, despite theoretical models of QoL indicating interactions (Mahjoob et al., 2024); this should be considered in future quantitative studies. To remedy this, theoretical and practical resources to explain and support good autistic QoL must be co-developed with the autistic community. Specifically, the evidence base lacks a comprehensive, community-led framework of autistic QoL that sets out (a) what QoL means to autistic people, (b) what constitutes good versus poor autistic QoL and (c) the key considerations of what promotes and/or impedes good QoL. Such a framework could inform supports by identifying appropriate, autistic-affirming outcome targets and processes (e.g., establishing positive connections and passionate interests instead of ‘making eye contact’ and ‘quiet hands’).

### Strengths, limitations and future work

Gathering the viewpoints of both autistic people and those supporting autistic children allowed for data triangulation (Carter et al., 2014). The strong concordance between factors raised across our two participant groups bolsters the trustworthiness of our findings. This is particularly noteworthy given that autistic adults and parents of autistic people often hold divergent perspectives (Rottier & Gernsbacher, 2020). Nevertheless, there are also limitations to our research. Most of our participants were White and well educated. The autistic adults who participated all used speech as their primary mode to communicate and reported no intellectual disabilities. In addition, since we recruited through social media and partner organisation networks, the online registration form required respondents to have internet access. Our findings must, therefore, be considered with these participant characteristics in mind. Furthermore, data from each informant group had unique limitations. Autistic adults provided retrospective data, drawing upon experiences that occurred in their childhood, which may have been decades prior. Parents of autistic children described current (and/or recent) experiences, but from an observer’s viewpoint, while parents of autistic adolescents and young adults spoke both retrospectively and from an observer’s viewpoint. Future work should, therefore, seek the views of autistic young people themselves, using methods and processes that enable them to share their views through their preferred means (Epstein et al., 2019).

We also acknowledge the many intersectional identities and individual circumstances that may shape each autistic child’s pathway to good QoL. Further work should consider whether the factors leading to good QoL differ with other elements of child identity (e.g., gender, race/ethnicity, profile of strengths and challenges) or family (socioeconomic status, geographic location) characteristics. All of this would help move from the basic question, ‘What helps autistic children to have a good QoL?’ to the more nuanced question, ‘What helps autistic children to have a good QoL, and for whom?’ This perspective is critical for informing the design of inclusive, responsive and neurodiversity-affirming services and professional practices that are attuned to the diverse realities of autistic children and their families. Our findings demonstrate that conventional QoL measures fail to consider factors that may be relevant – or, indeed, crucial – to supporting autistic children’s QoL. As a result of reliance on measures that overlook fundamental elements of autistic QoL, the existing evidence base may represent a considerable under-, over- or mis-estimate of autistic people’s true QoL. As a baseline, the findings from this study identified that supporting autistic children’s QoL involves accepting the autistic child, promoting personal connections in a way that is meaningful for them, providing the opportunity for them to pursue their interests, giving the child agency in their life and facilitating access to safe and supportive environments.

## Supplemental Material

sj-docx-1-aut-10.1177_13623613261418945 – Supplemental material for Supporting a ‘good life’ for autistic children: Autistic adults’ and parents’ perspectives Supplemental material, sj-docx-1-aut-10.1177_13623613261418945 for Supporting a ‘good life’ for autistic children: Autistic adults’ and parents’ perspectives by Kate Simpson, Connie Allen, Elizabeth Wheeley, Elizabeth Pellicano, Melanie Heyworth, Jacquiline den Houting, Rachael Bowen and Dawn Adams in Autism
