# Supplementary material for: Supporting a ‘good life’ for autistic children: Autistic adults’ and parents’ perspectives
Source: Autism. 2026 Feb 18;30(4):972–82. doi: 10.1177/13623613261418945 (PMC13005894; doi:10.1177/13623613261418945)
Supplement: sj-docx-1-aut-10.1177_13623613261418945 – Supplemental material for Supporting a ‘good life’ for autistic children: Autistic adults’ and parents’ perspectives [file sj-docx-1-aut-10.1177_13623613261418945.docx]

**Supplementary material**

**“Living a good life best life”: What helps or hinders the wellbeing of autistic children?**

**Autistic Adult Interview Schedule**

Hi, my name’s (Interviewer’s name), and I’m a researcher on this project…

Autistic people – just like everyone – deserve to live good lives, amazing lives even. But what makes a good life can mean different things to different people.

In this project, we want to understand what a good life means for young autistic children from your perspective – now and in the future. We also want to know what things might get in the way of them achieving a good life, as well as what might help them thrive. You can draw from your own childhood experience, or from things you have observed for other autistic children more recently.

We’re very grateful that you’ve chosen to meet with us and share a bit about the factors that you think can influence whether young autistic children can live their best life.

As you know, I am going to begin by asking you what you think a good life means for children today. And then we have FIVE different topics we’d like to focus on – you can talk about them in any order that you like.

Sometimes I may ask you to say more about what you’ve said, to get a clearer idea of the things you’re telling me. Please answer at your own pace, as there’s no rush. You can skip a question, or come back to a question at any time.

You can also stop or take a break any time. You can tell me you need a break, or you can turn off your camera and take a break if you prefer, or you can write that you need a break in the chat – whatever works for you.

If you need to move your body in any way to be comfortable, then please do.

I will also ask you during the interview if you’d like to take a rest. Also, please ask me to repeat or re-word a question, if you need to.

Also, please be assured that your responses will be anonymous and confidential, so in this project, your name will be replaced with a unique study number. The only exception to this is if something comes up that I think suggests a real risk of harm to you or to someone else, so I may need to inform relevant people, or give you information about support services.

Does that all sound ok?

As you know, we would like to record this interview so that it can be written up later. Is that still ok? Great – thank you.

Do you have any questions, before we start?

Ok, let’s get started!

**Start the Interview**

1. **ABOUT YOU**

Let’s start by talking about you. Tell me a bit about yourself. What brings you joy? What kinds of things do you find a bit more challenging? Was that similar when you were younger? To begin, can you think back of a time in your childhood when you felt able to live a “good life”. What things do you think were helped you to feel that way?

1. A **GOOD LIFE**

Now we will move on to think about specific topics and how they link to wellbeing of autistic children. Which topic would you like to start with?

As I mentioned, a good life can mean different things to different people (there’s no right or wrong answer!). And it has many different dimensions to it, too.

So, we’re going to try to look at what it means for autistic children to have a good life: by asking you to reflect on what you think a “good” life looks like for autistic children in different areas.

To help to structure our conversation, we are going to look at five key areas and ask the same questions for each one of those areas. The questions are:

(i) In this area, what does a good life look like for autistic children?

(ii) What obstacles stand in the way of achieving that good outcome? Or what sorts of things make it harder for autistic children to achieve a good outcome in this area?

(iii) Do you know of, or can you imagine, any ways of reducing or removing some of those obstacles or barriers?

You can choose which aspect we start with.

HAVING FUN: Think here about things that autistic children may do for fun, any interests/hobbies they have or leisure activities they are involved in. These can be at home or in the community. [maps on to play (leisure time/participation), other species in Capabilities Approach]

STAYING HEALTHY: Think here about what a healthy lifestyle may be for autistic children; think about nutrition/exercise, physical health and mental health [maps on to bodily integrity and emotion in Capabilities Approach]

HAVING A SAY, FEELING IN CONTROL: Think here about your autistic children’s identity, self-esteem, their confidence, their ability to plan for the future and shape what goes on around them [maps on to practical reason, control over one’s environment (and autonomy more broadly) in Capabilities Approach]

FAMILY AND FRIENDS: Think here about how autistic children relates to their parents, siblings, or their more extended family. Think also about how they relate to their friends and peers, like those from school or the neighbourhood [maps on to affiliation and bodily integrity in Capabilities Approach]

LEARNING. Think here about how autistic children learn and when they learn. You might think about the times they learn best or the conditions that help them to learn. Remember, learning can happen formally and informally, in the classroom or from you as parents, or with other important people in their life, or from books, TV and the internet [maps on to practical reason, senses, imagination and thought, and play in Capabilities Approach]

FINAL QUESTION: Almost done! If you could wave a magic wand to make a good life for your autistic child or young autistic people what one thing would you change? Is there anything else you think it’s important that I should know?

**Next steps**

That’s all my questions. Thanks so much for speaking with me and for sharing your experiences.

Regarding next steps, we are going to send the recording of your interview to a transcription service, who will write it out. And I will send you the transcript (the written-out version) of your interview in a few weeks’ time for you to review – to look over and make sure that you are happy with everything being included (and if you’re not, that’s fine, you can delete bits!). This step is optional, we know some people don’t like reading over what they’ve said, and that’s OK too.

Finally, I hope that you enjoyed our conversation. But if you feel worried or upset as a result of what we’ve spoken about today, please get in touch with your GP. I’ve also included the phone numbers for psychological support services and anonymous help-lines (beyondblue and Lifeline), in case you need to speak to someone anonymously about any concerns you have following this interview.

Thanks so much again and I look forward to being in touch again soon.

**Living a good life: What does that mean for your autistic child?**

**Parent Interview Schedule**

Hi, my name’s (Interviewer’s name), and I’m a researcher on this project.

Autistic people – just like everyone – deserve to live good lives, amazing lives even. But what makes a good life can mean different things to different people.

In this project, we want to understand what a good life means for your young autistic child from your perspective – now and in the future. We also want to know what things might get in the way of them achieving a good life, as well as what might help them thrive.

We’re very grateful that you’ve chosen to meet with us and share a bit about you and your family’s lives and the things that matter to you and your child.

As you know, I am going to begin by asking you what you think a good life means for your autistic child. And then we have FIVE different topics we’d like to focus on – you can talk about them in any order that you like.

Sometimes I may ask you to say more about what you’ve said, to get a clearer idea of the things you’re telling me. Please answer at your own pace, as there’s no rush. You can skip a question or come back to a question at any time.

You can also stop or take a break any time. You can tell me you need a break, or you can turn off your camera and take a break if you prefer, or you can write that you need a break in the chat – whatever works for you.

If you need to move your body in any way to be comfortable, then please do.

I will also ask you during the interview if you’d like to take a rest.

Also, please ask me to repeat or re-word a question, if you need to. Also, please be assured that your responses will be anonymous and confidential, so in this project, your name will be replaced with a unique study number. The only exception to this is if something comes up that I think suggests a real risk of harm to you or to someone else, so I may need to inform relevant people, or give you information about support services.

Does that all sound ok?

As you know, we would like to record this interview so that it can be written up later. Is that still ok? Great – thank you.

Do you have any questions, before we start?

Ok, let’s get started!

**Start the Interview**

Let’s start by talking a bit about your child and family.

1. **ABOUT YOUR FAMILY**

Tell me a bit about your family. Who do you live with (people and pets!)? How many children do you have, how old are they? How many of them are autistic?

Tell me more about [Autistic child’s name]. What kind of a person are they? What does a typical day look like for them? What kinds of things are they interested in/bring them joy? What kinds of things do they find difficult?

1. **A GOOD LIFE**

As I mentioned, a good life can mean different things to different people (there’s no right or wrong answer!). And it has many different dimensions to it, too.

So, we’re going to try to look at what it means for your Autistic child to have a good life: by asking you to reflect on what you think a “good” life looks like for your child in different areas.

To help to structure our conversation, we are going to look at five key areas and ask the same questions for each one of those areas. The questions are:

1. In this area, how does [Autistic child’s name] live a good life?
2. What obstacles stand in the way of achieving that good outcome? Or what sorts of things make it harder for [Autistic child’s name] to achieve a good outcome in this area?
3. Do you know of, or can you imagine, any ways of reducing or removing some of those obstacles or barriers?

You can choose which aspect we start with.

**LEARNING**. Think here about how your child learns and when they learn. You might think about the times they learn best or the conditions that help them to learn. Remember, learning can happen formally and informally, in the classroom or from you as parents, or with other important people in their life, or from books, TV and the internet [maps on to practical reason, senses, imagination and thought, and play in Capabilities Approach]

**FAMILY AND FRIENDS**: Think here about how your child relates to you (their parents) and their siblings, to their more extended family, and to their friends and peers, like those from school or the neighbourhood [maps on to affiliation and bodily integrity in Capabilities Approach]

**HAVING FUN**: Think here about things your child does for fun, any interests/hobbies they have or leisure activities they are involved in. These can be at home or in the community. [maps on to play (leisure time/participation), other species in Capabilities Approach] STAYING HEALTHY: Think here about how healthy [Autistic child’s name’s] lifestyle is, think about nutrition/exercise, physical health and mental health [maps on to bodily integrity and emotion in Capabilities Approach]

**HAVING A SAY, FEELING IN CONTROL**: Think here about your child’s identity, self-esteem, their confidence, their ability to plan for the future and shape what goes on around them [maps on to practical reason, control over one’s environment (and autonomy more broadly) in Capabilities Approach]

**FINAL QUESTION**: Almost done! If you could wave a magic wand to make a good life for your autistic child or young autistic people what one thing would you change?

Is there anything else you think it’s important that I should know?

**Next steps** That’s all my questions. Thanks so much for speaking with me and for sharing your experiences.

Regarding next steps, we are going to send the recording of your interview to a transcription service, who will write it out. And I will send you the transcript (the written-out version) of your interview in a few weeks’ time for you to review – to look over and make sure that you are happy with everything being included (and if you’re not, that’s fine, you can delete bits!). This step is optional, we know some people don’t like reading over what they’ve said, and that’s OK too.

Finally, I hope that you enjoyed our conversation. But if you feel worried or upset as a result of what we’ve spoken about today, please get in touch with your GP. I’ve also included the phone numbers for psychological support services and anonymous help-lines (beyondblue and Lifeline), in case you need to speak to someone anonymously about any concerns you have following this interview.

Thanks so much again and I look forward to being in touch again soon.
